# Compact SnO_2_/Mesoporous TiO_2_ Bilayer Electron Transport Layer for Perovskite Solar Cells Fabricated at Low Process Temperature

**DOI:** 10.3390/nano12040718

**Published:** 2022-02-21

**Authors:** Junyeong Lee, Jongbok Kim, Chang-Su Kim, Sungjin Jo

**Affiliations:** 1 School of Energy Engineering, Kyungpook National University, Daegu 41566, Korea; junyeong112@knu.ac.kr; 2 Department of Materials Science and Engineering, Kumoh National Institute of Technology, Gumi 39177, Korea; jbkim@kumoh.ac.kr; 3 Department of Advanced Functional Thin Films, Surface Technology Division, Korea Institute of Materials Science, Changwon 51508, Korea; cskim1025@kims.re.kr

**Keywords:** compact SnO_2_, mesoporous TiO_2_, oxygen plasma, perovskite solar cell low process temperature

## Abstract

Charge transport layers have been found to be crucial for high-performance perovskite solar cells (PSCs). SnO_2_ has been extensively investigated as an alternative material for the traditional TiO_2_ electron transport layer (ETL). The challenges facing the successful application of SnO_2_ ETLs are degradation during the high-temperature process and voltage loss due to the lower conduction band. To achieve highly efficient PSCs using a SnO_2_ ETL, low-temperature-processed mesoporous TiO_2_ (LT m-TiO_2_) was combined with compact SnO_2_ to construct a bilayer ETL. The use of LT m-TiO_2_ can prevent the degradation of SnO_2_ as well as enlarge the interfacial contacts between the light-absorbing layer and the ETL. SnO_2_/TiO_2_ bilayer-based PSCs showed much higher power conversion efficiency than single SnO_2_ ETL-based PSCs.

## 1. Introduction

Perovskite solar cells (PSCs) have received attention because their power conversion efficiency (PCE) has rapidly increased by over 25% [1,2]. Many researchers have tried to enhance the performance of PSCs and translate them from the laboratory to commercial products [3,4,5,6].

For the state-of-the-art device configuration, PSCs usually consist of a transparent electrode, an electron transport layer (ETL), a light-absorbing layer, a hole transport layer (HTL), and a metal electrode [7]. In the pursuit of high-performance PSCs, the ETL has become the subject of high interest and one of the most challenging scientific issues [8]. As a conventional ETL material, TiO_2_ has been widely adopted. However, many attempts have been made to substitute TiO_2_ with alternative materials that have better optoelectronic properties [9]. SnO_2_ is the most investigated ETL after TiO_2_ due to its high electron mobility, high conductivity, wide optical bandgap, and excellent chemical stability [10]. Although SnO_2_ ETL-based PSCs have made rapid progress recently, their performance is still lower than that of PSCs using mesoporous TiO_2_ (m-TiO_2_) as an ETL [11]. In addition, the low conduction band of SnO_2_ reduces the built-in potential of the Schottky barrier between the perovskite and SnO_2_, resulting in the voltage loss of the PSCs [12].

To achieve highly efficient PSCs using a SnO_2_ ETL, SnO_2_ ETL combination and surface modification techniques that can improve electron injection and suppress electron recombination have been developed [13]. Various inorganic metal oxides, such as ZnO [14], MgO [15], and TiO_2_ [16,17], as well as organics, including carbon-based materials [18], self-assembled monolayers (SAM) [19], and polymers [20], have been adopted in SnO_2_ ETL-based PSCs to combine with or modify SnO_2_. Among them, the conventional m-TiO_2_ layer is the preferable candidate to be combined with the compact SnO_2_ (c-SnO_2_) layer because the mesoporous scaffold can facilitate sufficient pore filling of the light-absorbing layer and improve electron extraction and transport over a single c-SnO_2_ layer [17,21]. Moreover, TiO_2_ is a better choice in view of its established cascaded energy-level alignment between the electrode and light-absorbing layer, which results in a significantly improved performance. However, m-TiO_2_ generally requires a high-temperature sintering process of up to 450 °C to remove organic additives that cause deterioration in the photovoltaic performance [22]. This high-temperature process restricts the application of m-TiO_2_ on the c-SnO_2_ layer because the high-temperature process induces not only a large amount of charge traps and a recombination center in the SnO_2_ layer but also poor interfacial contact, leading to interface recombination and shunting paths. Therefore, one important challenge is determining how to construct m-TiO_2_ on the c-SnO_2_ layer to take advantage of SnO_2_. We recently achieved low-temperature processed PSCs by employing m-TiO_2_ as ETL [23]. To remove the organic additives in the low-temperature-processed TiO_2_ (LT-TiO_2_), we adopted the oxygen plasma process. The simple and effective method of oxygen plasma treatment enhances charge extraction and transport, thereby improving photovoltaic performance. Therefore, our newly developed oxygen plasma treatment for LT m-TiO_2_ is a promising strategy for combining the m-TiO_2_ layer with the c-SnO_2_ layer to produce an efficient bilayer ETL.

In this work, we demonstrated that the LT m-TiO_2_ can be adopted to construct a compact/mesoporous structured bilayer ETL to prevent the degradation of SnO_2_ by the high-temperature process. When the conventional m-TiO_2_ layer was deposited on the SnO_2_ layer and then the bilayer ETL underwent the high-temperature sintering process (BLH), the photovoltaic performance of this bilayer ETL-based PSC (BLH-PSC) deteriorated more than that of a single c-SnO_2_ ETL-based PSC (SL-PSC). On the contrary, when the oxygen plasma treatment was applied to the LT m-TiO_2_ deposited on the SnO_2_ (BLP), the PSC with this bilayer ETL (BLP-PSC) exhibited an excellent PCE of 15.36%, which is higher than that of the SL-PSC (13.68%). Moreover, detailed characterizations demonstrated that the SnO_2_/TiO_2_ bilayer ETL is beneficial for carrier extraction and transport.

## 2. Materials and Methods

### 2.1. Fabrication of the SnO_2_ Layer

A fluorine-doped tin oxide (FTO) electrode was patterned using zinc powder and diluted HCl solution. Then, the patterned FTO substrate was cleaned with deionized water (DI), acetone, and ethanol in an ultrasonic bath. After ultraviolet–ozone (UVO) treatment for 15 min, 0.05 M SnCl_2_·2H_2_O solution diluted in ethanol was spin-coated on the patterned FTO substrate and sintered at 200 °C for 1 h.

### 2.2. Fabrication of the Bilayer ETL

The m-TiO_2_ solution was prepared by dissolving TiO_2_ nanoparticle paste (Dyesol, Queanbeyan, Australia) in ethanol at a ratio of 1:10 (wt %). After 15 min of UVO treatment, the m-TiO_2_ solution was spin-coated on the SnO_2_ layer and sintered at 150 °C for 4 h for BLL-PSC and BLP-PSC. To remove TiO_2_ nanoparticle aggregates, the bilayer ETL substrate was dipped in ethanol and stirred for 15 s, and then the substrate was annealed at 150 °C for 30 min. In contrast, the m-TiO_2_ layer was sintered at 450 °C for 1 h and was not rinsed with ethanol for BLH-PSC. The substrate was dipped in 20 mM TiCl_4_ solution at 90 °C for 15 min and sintered at 150 °C for 30 min.

### 2.3. Fabrication of the PSC

The MAPbI_3_ solution was prepared by mixing methylammonium iodide (MAI), PbI_2_, dimethyl sulfoxide, and *N*,*N*-dimethylformamide. In the case of BLP-PSCs, the m-TiO_2_ layer was treated by oxygen plasma at a radio frequency (RF) power of 20 W for 10 min. Then, the MAPbI_3_ layer was spin-coated and annealed at 65 °C for 1 min and at 100 °C for 10 min. The mixed Spiro-OMeTAD solution, which contained Spiro-OMeTAD (Jilin OLED, Changchun, Jilin Sheong, China), lithium salt, 4-tert-butylpyridine, and chlorobenzene, was spin-coated on the MAPbI_3_ layer. A silver electrode was deposited via a thermal evaporator. All chemicals were purchased from Sigma-Aldrich (St. Louis, MO, USA).

### 2.4. Fabrication of the Flexible PSC

The indium-doped tin oxide (ITO)/polyethylene naphthalate (PEN) substrate (Peccell Technologies, Yokohama, Japan) was used to fabricate flexible PSCs. All fabrication processes were identical to that for BLP-PSC on FTO substrate, only the SnO_2_ layer was annealed at 150 °C for 5 h.

### 2.5. Measurements

The surface morphologies were characterized using a field-emission scanning electron microscope (SEM) (S-4800, HITACHI, Tokyo, Japan). The photovoltaic characteristics were measured under 100 mW/cm^2^ illumination using a solar simulator (Sol2A, Oriel, Irvine, CA, USA) with scan rate of 0.02 V at 25 °C. The internal electrochemical behavior was characterized using electrochemical impedance spectroscopy (EIS) (Compactstat.h, Ivium Technologies, Eindhoven, Netherlands) at a frequency range of 1 Hz to 1 MHz. The bending test was performed at a rate of 1 cycle per 0.5 s and a bending radius of 13 mm using a radius bending tester (JIRBT-620, JUNIL TECH, Daegu, Korea).

## 3. Results and Discussion

**Figure 1:**
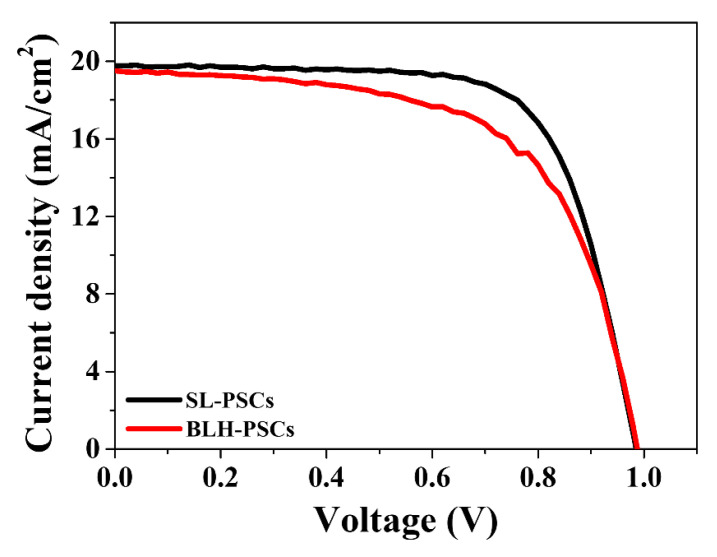
Reverse scan current density–voltage (J-V) curves of SL-PSC and BLH-PSC.

To investigate the possibility of using the conventional high-temperature-processed m-TiO_2_ layer as the layer combined with the c-SnO_2_ layer, we performed a comparative study of PSCs using both planar- and mesoporous-type PSCs. SL-PSCs in the configuration of FTO/c-SnO_2_/perovskite/spiro-OMeTAD/Ag and BLH-PSCs in the configuration of FTO/c-SnO_2_/m-TiO_2_/spiro-OMeTAD/Ag were fabricated. In the case of the BLH-PSCs, the m-TiO_2_ layer was sintered at 450 °C after spin-coating on the SnO_2_ layer. The current density–voltage (J-V) curves under an irradiation of 100 mW cm^−2^ (AM 1.5) are shown in Figure 1. The SL-PSCs achieved a PCE of 13.68% with an open-circuit voltage (V_OC_) of 0.99 V, a short-circuit current density (J_SC_) of 19.77 mA/cm^2^, and a fill factor (FF) of 70.22%. In contrast, the BLH-PSCs only achieved a PCE of 11.93% with a V_OC_ of 0.99 V, a J_SC_ of 19.52 mA/cm^2^, and an FF of 61.86%. It is thus clear that SL-PSCs perform much better than BLH-PSCs. The large difference in FF could be primary attributed to the degradation of the SnO_2_ layer by the high-temperature process [22].

**Figure 2:**
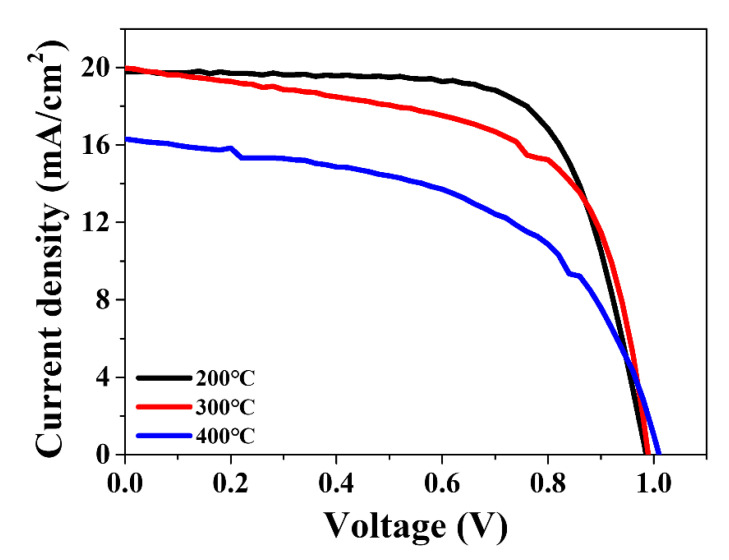
Reverse scan current density–voltage (J-V) curves of perovskite solar cells based on SnO_2_ ETL annealed at 200, 300, and 400 °C.

To uncover the underlying reasons for the decreased photovoltaic performance of BLH-PSCs, we fabricated SL-PSCs using a SnO_2_ ETL annealed at temperatures from 200–500 °C. Figure 2 shows the dependence of PCE on the annealing temperature of the SnO_2_ layer. As the annealing temperature increased, the photovoltaic performance of SL-PSCs decreased. The PECs of SL-PSCs annealed at 200, 300, and 400 °C were 13.68%, 12.19%, and 8.90%, respectively. The SL-PSCs annealed at 400 °C performed poorly, with very low FF and J_SC_. Moreover, the SL-PSCs annealed at 500 °C did not show any photovoltaic characteristics. The detailed photovoltaic parameters obtained from the J-V curves are summarized in Table S1.

**Figure 3:**
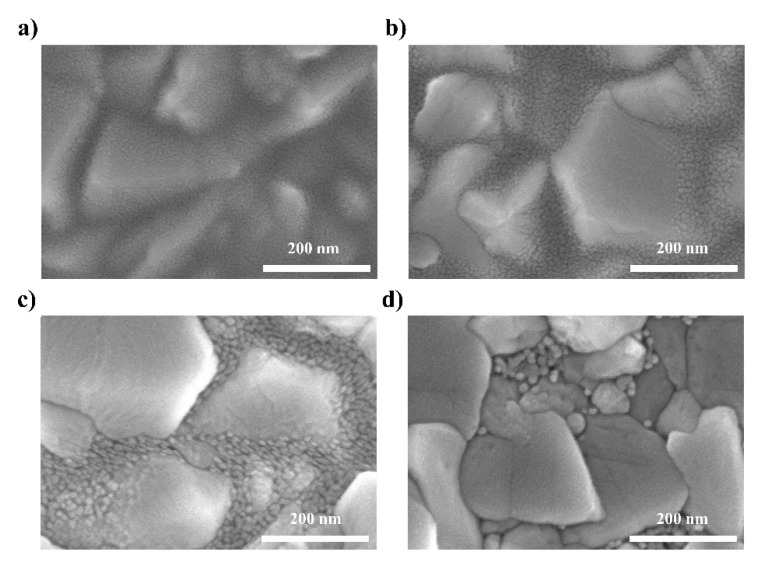
Top SEM image of SnO_2_ layer after annealing at (**a**) 200, (**b**) 300, (**c**) 400, and (**d**) 500 °C on FTO glass.

To verify the decreasing trend in PCE, we investigated the morphology change of the SnO_2_ layer according to the annealing temperature. Figure 3 shows the top-view SEM images of SnO_2_ layers deposited on FTO substrates and annealed at different temperatures. As shown in Figure 3a, the FTO substrate was uniformly covered with the SnO_2_ layer, and no pinholes were observed when the SnO_2_ layer was annealed at the relatively low temperature of 200 °C. However, as the annealing temperature increased above 300 °C, the SnO_2_ nanoparticles agglomerated more and the FTO areas uncovered by SnO_2_ increased. The high-temperature-annealed SnO_2_ layer could not completely cover the FTO substrate, and thus these pinholes resulted in leakage in the current pathway. Moreover, poor interface contact with the FTO substrate increased the series resistance. Therefore, as shown in Figure 1, BLH-PSCs exhibited lower FF and PCE than SL-PSCs because the high-temperature process of the m-TiO_2_ layer caused the degradation of the underlying SnO_2_ layer [24]. The above results confirm that the low-temperature processing of the m-TiO_2_ layer without causing damage to the SnO_2_ layer is important for producing high-performance PSCs using a c-SnO_2_/m-TiO_2_ bilayer ETL.

**Figure 4:**
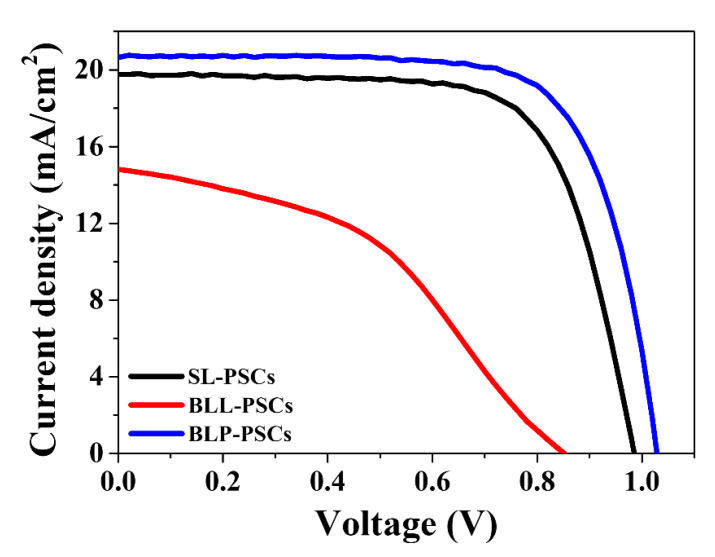
Reverse scan current density–voltage (J-V) curves of SL-PSCs, BLL-PSCs, and BLP-PSCs.

To investigate the effectiveness of our strategy for LT m-TiO_2_, we compared the PCEs of three different PSCs (Figure S2). First, we fabricated SL-PSC without the m-TiO_2_ layer to determine the role of the m-TiO_2_ layer. Then, we fabricated two different PSCs based on the FTO/c-SnO_2_/m-TiO_2_/spiro-OMeTAD/Ag architecture. The main difference between these two PSCs with a bilayer ETL was the post-treatment of the m-TiO_2_ layer. In the case of LT m-TiO_2_-based PSCs (BLL-PSCs), the m-TiO_2_ layers were only annealed at 150 °C, whereas oxygen plasma treatment was directly performed on the LT TiO_2_ layers for BLP-PSCs. As shown in Figure 4, the BLL-PSCs exhibited a PCE of only 5.43% with a V_OC_ of 0.85 V, a J_SC_ of 14.81 mA/cm^2^, and an FF of 43.01% (Table 1). Although the low-temperature processing of m-TiO_2_ might not have caused the aggregation of the underlying c-SnO_2_ layer, the remaining organic additives in m-TiO_2_ inhibited the full coverage of the perovskite layer on TiO_2_, which hindered electron transport at the interface between the perovskite and TiO_2_. According to our previous work, oxygen plasma treatment can successfully remove organic additives from and improve the wettability of the LT TiO_2_ layer [13]. With oxygen plasma treatment, the performance of BLL-PSCs considerably improved, and the PCE, V_OC_, J_SC_, and FF were 15.35%, 1.03 V, 20.65 mA/cm^2^, and 72.30%, respectively. Moreover, the PCE of BLP-PSC (15.53%) is higher than that of SL-PSC (13.68%). These results demonstrate that oxygen plasma treatment enables the fabrication of c-SnO_2_/m-TiO_2_ bilayer ETL-based PSCs with excellent photovoltaic performance using an LT m-TiO_2_ layer.

**Figure 5:**
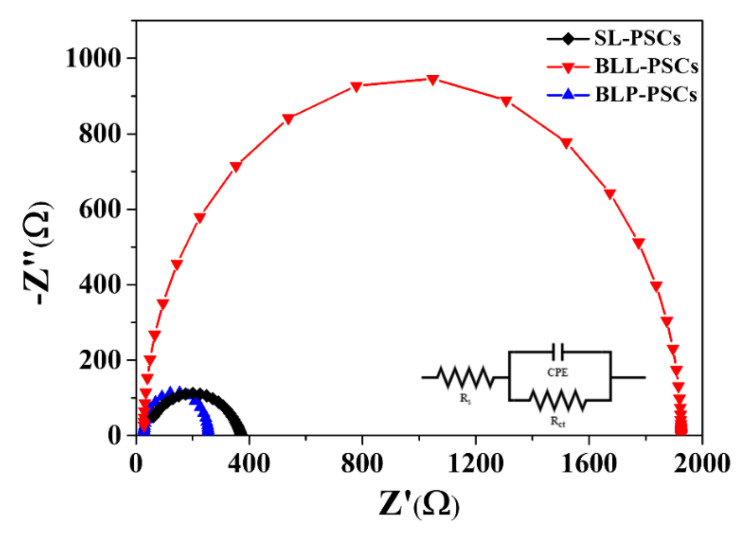
Nyquist plots of SL-PSCs, BLL-PSCs, and BLP-PSCs.

To gain further insight into the effects of the m-TiO_2_ layer on charge transfer properties at the ETL/perovskite interface, EIS was conducted. The Nyquist plots of different ETLs were obtained in the dark with an applied bias voltage of 0.9 V and are shown in Figure 5. The series resistance (R_s_) and charge transport resistance (R_ct_) were obtained by fitting EIS data according to the relevant equivalent circuit, as shown in the inset of Figure 5. The EIS parameters from the semicircle Nyquist plot are summarized in Table S2. In general, R_s_ is related to the sheet resistance of electrodes [25], including the contributions from FTO and metal electrodes. In contrast, R_ct_ generally refers to the charge transfer resistance at all the interfaces [26], such as between the carrier selective layer and the perovskite layer, and between the electrode and the carrier selective layer. The R_ct_ value of SL-PSC was 350 Ω, which is slightly higher than that of BLP-PSC (230 Ω). The small R_ct_ value of BLP-PSC further supports that the combination of the m-TiO_2_ layer with c-SnO_2_ promotes good interface contact between the ETL and perovskite, leading to an enhanced charge transfer process and the highest PCE [27]. While BLL-PSC exhibited the highest value of R_ct_, it had the lowest PCE. Figure S1 shows the top-view SEM images of the perovskite layer on c-SnO_2_ and c-SnO_2_/m-TiO_2_ and their corresponding grain size distribution histograms. Both perovskite layers exhibited a similar average grain size with uniform morphology consisting of densely packed grains. Because the grain size, which refers to the density of grain boundaries, is related to the transport of photogenerated carriers and the extension of the charge carrier diffusion length [28], a comparable grain size might not result in different photovoltaic characteristics. However, the mesoporous structure of TiO_2_ allows the perovskite to infiltrate into TiO_2_, which increases the interfacial contact between perovskite and TiO_2_ (Figure S3). Therefore, the improved interfacial contact due to the direct transfer pathway substantially contributed to the increase in PCE of SL-PSCs by combination with the m-TiO_2_ ETL (Figure S4 and Table S3).

**Figure 6:**
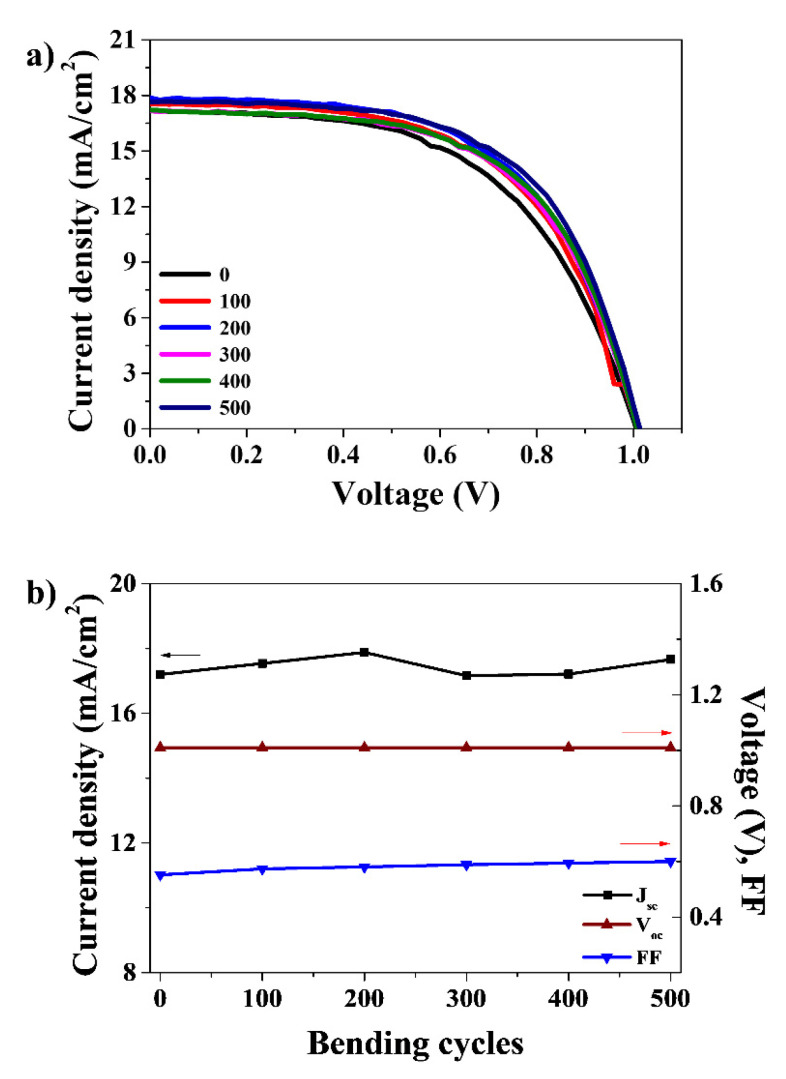
(**a**) Reverse scan current density–voltage (J-V) curves of flexible cells and (**b**) variation in the J_SC_, V_OC_, and FF values of flexible cells as a function of bending cycles.

The high performance of c-SnO_2_/m-TiO_2_ bilayer ETL-based PSCs was achieved with oxygen plasma treatment at a low temperature, which suggests that high-performance and flexible BLP-PSCs can also be attained via the same procedures. Figure 6 shows the J-V curves of the flexible BLP-PSCs constructed on the PEN/ITO substrate as a function of bending cycles. The flexible cells exhibited a promising PCE of 9.56%, with a V_OC_ of 1.81 V, a J_SC_ of 17.20 mA/cm^2^, and an FF of 55.22%. The inferior performance of the flexible cell compared to the rigid cell on the FTO/glass substrate arose from its inferior surface morphology and low transmittance compared to the rigid surface [29]. To investigate the mechanical stability of the flexible cell, a bending durability test was performed. All the photovoltaic characteristics were maintained during 500 cycles of bending without much deterioration.

## 4. Conclusions

In summary, an LT m-TiO_2_ layer using oxygen plasma treatment was combined with c-SnO_2_. The BLP-PSC had a PCE of 15.36%, which is much higher than that of the PSC with a single c-SnO_2_ ETL_._ This high efficiency was obtained because the oxygen plasma treatment facilitated the removal of organic additives from LT m-TiO_2_ and the infiltration of perovskite into m-TiO_2_, thus enhancing charge transport and extraction. This proves that our strategy to construct a bilayer ETL using LT m-TiO_2_ is beneficial because it allows the superior characteristics of the underlying c-SnO_2_ to be maintained, and the mesoporous structure provided increased interfacial contact between the perovskite and the ETL. Consequently, the c-SnO_2_/m-TiO_2_ bilayer ETL is thought to be one of the most promising ETL layers for high-efficiency PSCs.

## Figures and Tables

**Table 1:** Summary of the photovoltaic parameters of PSCs based on different ETLs.

Sample	J_SC_ (mA/cm^2^)	V_OC_ (V)	FF (%)	PCE (%)
SL-PSCs	19.77	0.99	70.22	13.68
BLL-PSCs	14.81	0.85	43.01	5.43
BLP-PSCs	20.65	1.03	72.30	15.36

## Data Availability

The data presented in this article is available on request from the corresponding author.

## Supplementary Materials

The following supporting information can be downloaded at: https://www.mdpi.com/article/10.3390/nano12040718/s1, Figure S1: Top-view SEM images and grain size distribution histograms (inset) of MAPbI_3_ layer on SL, BLL, BLP, and BLH; Figure S2: XRD pattern of MAPbI_3_ layer on BLP, BLL, and SL; Figure S3: Cross-section SEM image of glass/FTO/ETL/MAPbI_3_; Figure S4: Reverse scan (solid line) and forward scan (dotted line) current density–voltage curves of PSCs based on SL-PSC, BLL-PSC, and BLP-PSC; Table S1: Summary of the photovoltaic parameters of PSCs based on SnO_2_ ETL annealed at 200, 300, and 400 °C; Table S2: Summary of EIS data; Table S3: Summary of the photovoltaic parameters of SL-PSC, BLL-PSC, and BLP-PSC according to scan direction.
