# Supplementary material for: Compact SnO2/Mesoporous TiO2 Bilayer Electron Transport Layer for Perovskite Solar Cells Fabricated at Low Process Temperature
Source: Nanomaterials (Basel). 2022 Feb 21;12(4):718. doi: 10.3390/nano12040718 (PMC8875851; doi:10.3390/nano12040718)
Supplement: Supplementary file 1 [file nanomaterials-12-00718-s001.zip › nanomaterials-1583470-supplementary.pdf]

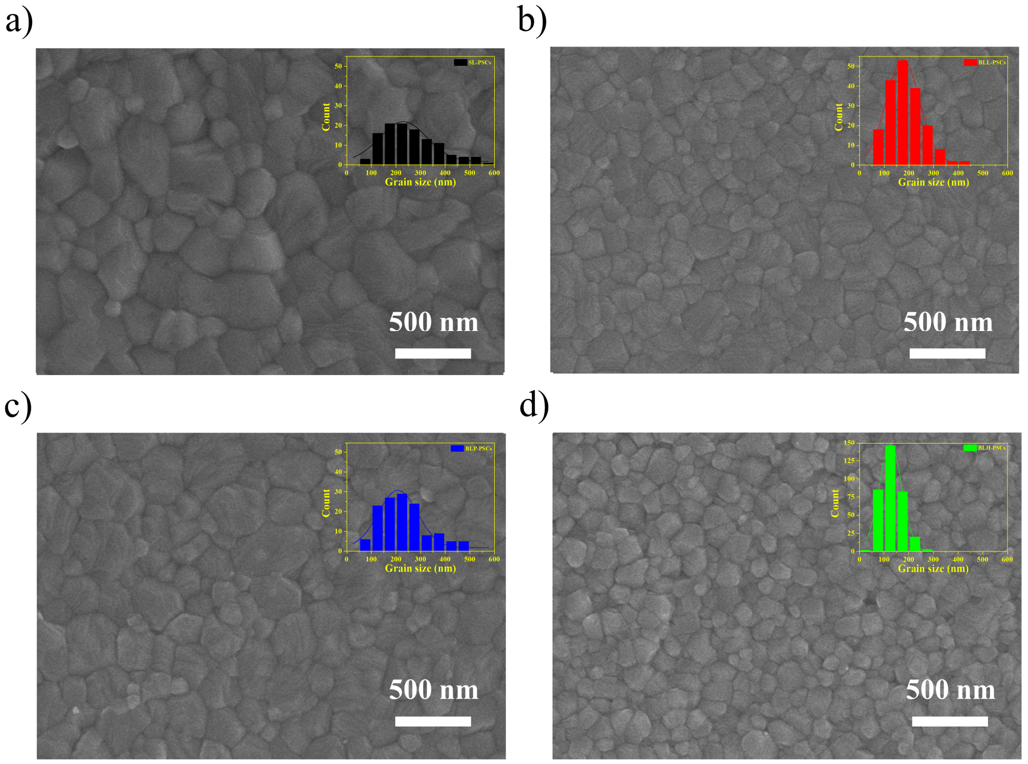

Figure S1. Top view SEM images and grain size distribution histograms (inset) of $MAPbI_3$ layer on (a) SL, (b) BLL, (c) BLP, and (d) BLH.

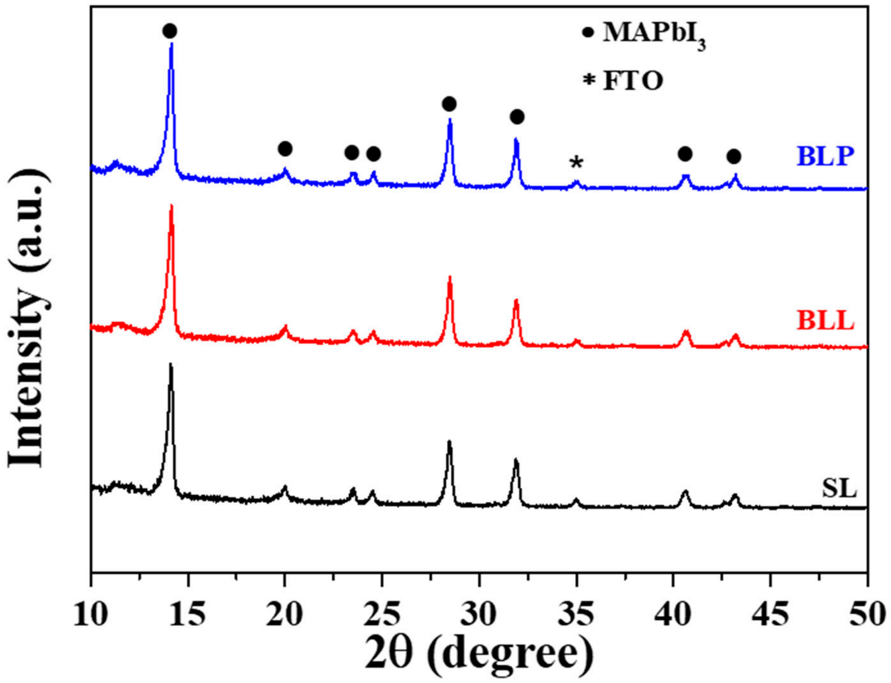

Figure S2. XRD pattern of $MAPbI_3$ layer on BLP, BLL, and SL.

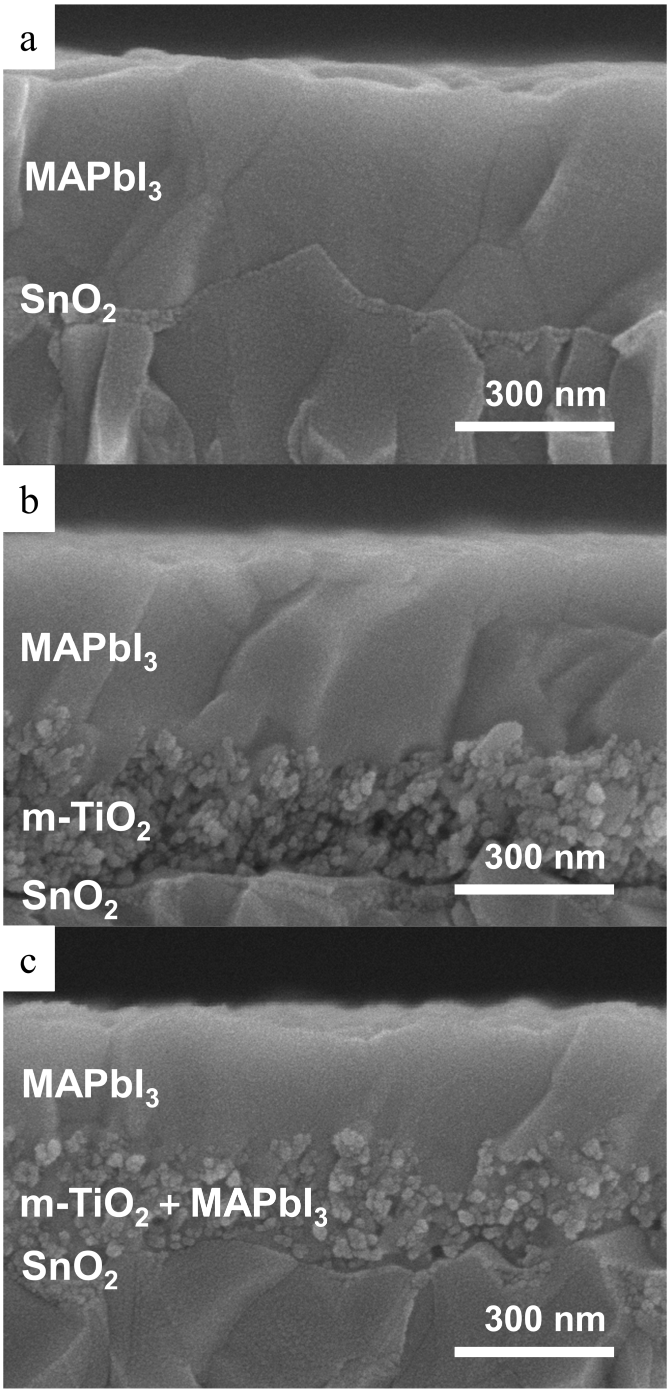

Figure S3. Cross-section SEM image of glass/FTO/(a) SL, (b) BLL, and (c) BLP/$MAPbI_3$.

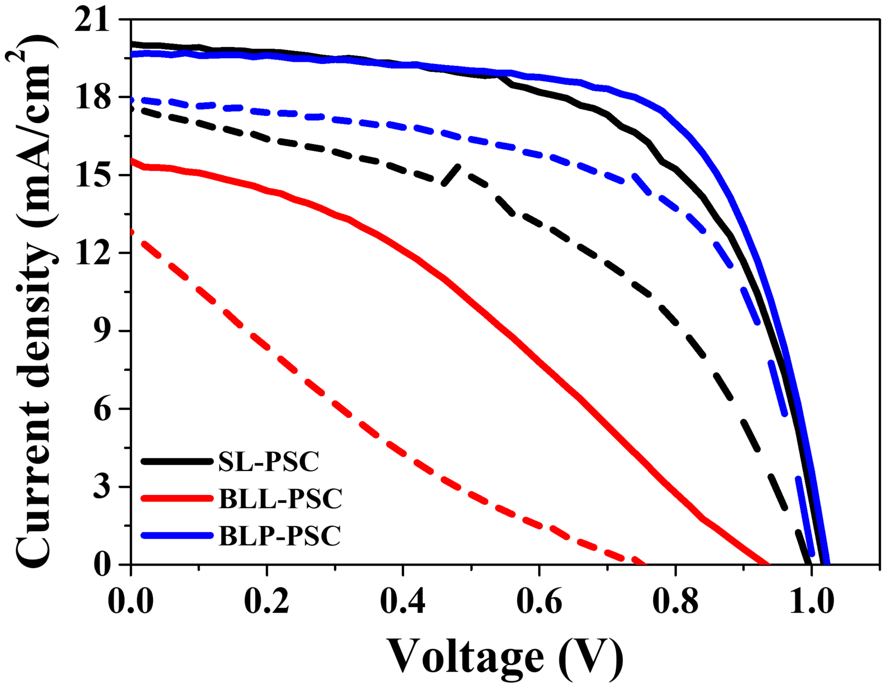

Figure S4. Reverse scan (solid line) and forward scan (dotted line) current density-voltage curves of SL-PSC, BLL-PSC, and BLP-PSC.

x

Table S1. Summary of the photovoltaic parameters of PSCs based on SnO_2 ETL annealed at 200℃, 300℃, and 400℃.

Temperature	J_{sc} (mA/cm^2)	V_{oc} (V)	FF (%)	PCE (%)
200℃	19.77	0.99	70.22	13.68
300℃	20.00	0.99	61.68	12.19
400℃	16.08	1.01	55.00	8.90

Table S2. Summary of EIS data.

Sample	R_s Value (Ω)	R_{ct} Value (Ω)
SL-PSC	27.13	350
BLL-PSC	27.85	1900
BLP-PSC	27.30	230

Table S3. Summary of the photovoltaic parameters of SL-PSC, BLL-PSC, and BLP-PSC according to scan direction.

Samples	J_{SC} (mA/cm^2)	V_{OC} (V)	FF (%)	PCE (%)	Hysteresis index
SL-PSCs (R)	20.18	1.03	61.81	12.80	0.39
SL-PSCs (F)	17.55	1.00	46.44	8.11	
BLL-PSCs (R)	15.54	0.93	34.99	5.07	0.65
BLL-PSCs (F)	12.80	0.75	19.35	1.86	
BLP-PSCs (R)	19.65	1.02	67.85	13.63	0.17
BLP-PSCs (F)	17.88	1.00	61.71	11.06	

* Hysteresis index = $\frac{J_{RS}(0.8V_{OC}) - J_{FS}(0.8V_{OC})}{J_{RS}(0.8V_{OC})}$
